# Crystal-Inspired Cellular Metamaterials and Triply Periodic Minimal Surfaces

**DOI:** 10.3390/biomimetics9050285

**Published:** 2024-05-10

**Authors:** Maxim Arsentev, Eduard Topalov, Sergey Balabanov, Evgenii Sysoev, Igor Shulga, Marsel Akhmatnabiev, Maxim Sychov, Ekaterina Skorb, Michael Nosonovsky

**Affiliations:** 1 Infochemistry Scientific Center (ISC), ITMO University, 9 Lomonosova St., St. Petersburg 191002, Russia; ars21031960@gmail.com (M.A.); skorb@itmo.ru (E.S.); 2 Institute of Silicate Chemistry, Russian Academy of Sciences, St. Petersburg 199034, Russiashulga_gosha@list.ru (I.S.); marsel.akhmatnabiev@mail.ru (M.A.); msychov@yahoo.com (M.S.); 3 Department of Micro- and Nanoelectronics, Saint Petersburg Electrotechnical University “LETI”, Professor Popov Str. 5, St. Petersburg 197376, Russia; 4 College of Engineering and Applied Science, University of Wisconsin-Milwaukee, Milwaukee, WI 53211, USA

**Keywords:** 3D printing, additive manufacturing, cellular materials, crystal structure

## Abstract

Triply periodic minimal surfaces (TPMSs) are found in many natural objects including butterfly wings, sea urchins, and biological membranes. They simultaneously have zero mean curvature at every point and a crystallographic group symmetry. A metamaterial can be created from such periodic surfaces or used as a reinforcement of a composite material. While a TPMS as a mathematical object has been known since 1865, only novel additive manufacturing (AM) technology made it possible to fabricate cellular materials with complex TPMS shapes. Cellular TPMS-based metamaterials have remarkable properties related to wetting/liquid penetration, shock absorption, and the absence of stress concentrators. Recent studies showed that TPMSs are also found in natural crystals when electron surfaces are considered. Artificial crystal-inspired metamaterials mimic such crystals including zeolites and schwarzites. These metamaterials are used for shock, acoustic waves, and vibration absorption, and as structural materials, heat exchangers, and for other applications. The choice of the crystalline cell of a material, as well as its microstructure, plays a decisive role in its properties. The new area of crystal-inspired materials has many common features with traditional biomimetics with models being borrowed from nature and adjusted for engineering applications.

## 1. Introduction

Metamaterials derive their properties not from the properties of the base materials but from their structures and patterns from which they are derived. In the present paper, we consider a specific type of metamaterial, triply periodic minimal surfaces (TPMSs). We discuss the relation of TPMSs to crystals and recent progress in the development of such materials.

TPMS metamaterials can be classified as nature-inspired materials due to their relation to naturally occurring crystals. Biomimetics is often defined as mimicking nature for engineering applications [1,2], and usually living nature is implied. The definition of the International Organization for Standardization (The Standard 18458 of 2015) distinguishes between *biomimetics* (“an interdisciplinary cooperation of biology and technology or other fields of innovation with the goal of solving practical problems through the function analysis of biological systems, their abstraction into models, and the transfer into and application of these models to the solution”), *bionics* (“a technical discipline that seeks to replicate, increase, or replace biological functions by their electronic and/or mechanical equivalents”), *biomimicry* or *biomimetism* (“philosophy and interdisciplinary design approaches taking nature as a model to meet the challenges of sustainable development”), and *bioinspiration* (“a creative approach based on the observation of biological systems”) [2,3].

Most biomimetic materials and surfaces use living nature as an inspiration. However, natural crystals, while non-living, may also serve as a source of inspiration for new materials. This is because for many solid crystalline compounds, zero-equipotential surfaces, electron density, and electron localization distribution functions take the form of triply periodic minimal surfaces (TPMSs) [4]. Consequently, crystal-inspired materials can constitute a novel class of biomimetic materials. It is noted also that besides crystals, TPMSs are also found in living nature including butterfly wings, sea urchins, and biological membranes [5]. The surfaces obtained by constructing the isoelectronic density or the surface of zero coulomb potential have the form of TPMSs [6]. TPMSs have many applications ranging from photonics to gas sensors [7,8,9,10,11,12,13].

A minimal surface minimizes its area for a given boundary contour, which is equivalent to possessing zero mean curvature, i.e., the sum of two principal curvatures at a point:

(1) $$H=\frac{1}{R_{1}}+\frac{1}{R_{2}}=0$$

**Figure 1:**
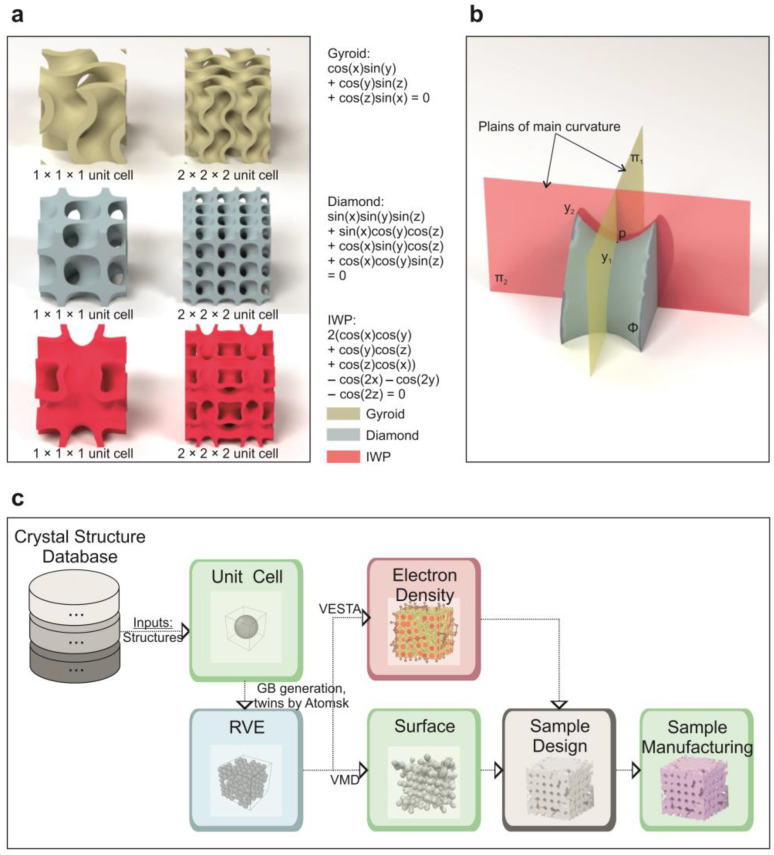
(**a**) Mathematical level-set approximations of the designed TPMS architectures; (**b**) rendering of saddle surface with zero mean curvature; (**c**) schematic representation of crystal-inspired cellular materials design. Representative volume element (RVE) corresponds to the minimum volume for the design of a cellular structure with or without grain boundaries (GBs), meta-precipitates, and multiphase-like metamaterials with enhanced mechanical performance. Atomsk v0.13 [34], VESTA 3.5.5 [33], and VMD 1.9.4 [32] are software tools.

Consequently, every point on a minimal surface is a saddle point, and the surface is neither convex nor concave. An everyday example of a minimal surface would be a soap film, formed by a soap bubble at a given boundary contour. The film minimizes its surface energy and thus attains the shape with a minimal possible surface area [14]. There are many types of TPMSs, and some of them are presented in Figure 1a. These surfaces minimize their area for a given boundary contour, which is equivalent to possessing zero mean curvature, i.e., the sum of two principal curvatures at a point (Figure 1b).

In addition to possessing zero mean curvature, a TPMS is invariant under a rank-3 lattice of translations and forms a spatial periodic cellular structure. Mathematicians have known such surfaces since the 1860s as pure geometrical objects (e.g., Schwarz minimal surfaces) [15]. Since that time, mathematicians have discovered many new types of TPMSs; however, simple methods to create TPMSs became available only with the advent of additive manufacturing (AM) and 3D-printing technologies.

The physical significance of TPMSs is twofold, and it is a consequence of two properties of minimal surfaces: controlled curvature and minimized surface area. Since many properties of surfaces, such as wetting properties and mechanical stress concentration, depend on their mean curvature, TPMSs have properties different from those of regular porous materials. For example, the penetration of water into porous materials is limited by cavities being convex or possessing negative mean curvature. A TPMS has zero curvature at every point, so water can penetrate easily. During mechanical loading, fracture tends to be initiated at regions of stress concentration, such as sharp angles or junctions. A TPMS has no sharp angles or junctions.

Moreover, since a TPMS minimizes surface area and, therefore, the amount of material, it is also expected that it may provide optimized properties per unit weight, such as strength per density. Such properties are needed for many applications; for example, stiff but light materials are needed for impact resistance [16]; thus, stiffness (elastic modulus) vs. density is a standard representation of materials in the so-called Ashby chart, which is considered later.

Lattice structures have a high strength-to-weight ratio and energy absorption. To design and identify materials that can withstand loads, impact resistance, and effective energy absorption, one can draw inspiration from examples of nature. Mechanical properties can be adapted through composition, geometry, and topology, focusing on the potential of AM technologies such as 3D printing.

The number of publications dedicated to TPMSs for metamaterials science is growing steadily and they appear in high-impact journals. The results described in the research papers are promising, but much remains to be unexplored. Since the physical and mechanical properties of metamaterials depend on the topological features of TPMSs, the classification of these surfaces and their topological exploration are important. TPMSs nowadays attract the attention of material scientists as promising models for new materials that can be synthesized by the 3D printing technique.

TPMSs are found in many objects of living nature, such as butterfly wings [17,18], sea urchins [19], and biological membranes [20], but research shows that they can be obtained on the basis of crystals; this approach is called the crystal-inspired approach. Considering the history of the crystal-inspired approach, it is worth mentioning the classic work of von Schnering et al. [6] in which a direct relationship is established between the symmetry of the TPMSs and the crystal structure that is capable of generating it. Additive manufacturing (AM) currently opens up the possibility of creating complex structures [21,22,23,24,25], namely, architectural cellular materials, often called lattice structures. These consist of periodic structures called elementary cells. By changing the parameters of these elementary cell structures, new functionality can be achieved in acoustic [26], electrical [27], thermal [28,29], magnetic [30], and optical properties [31].

**Figure 2:**
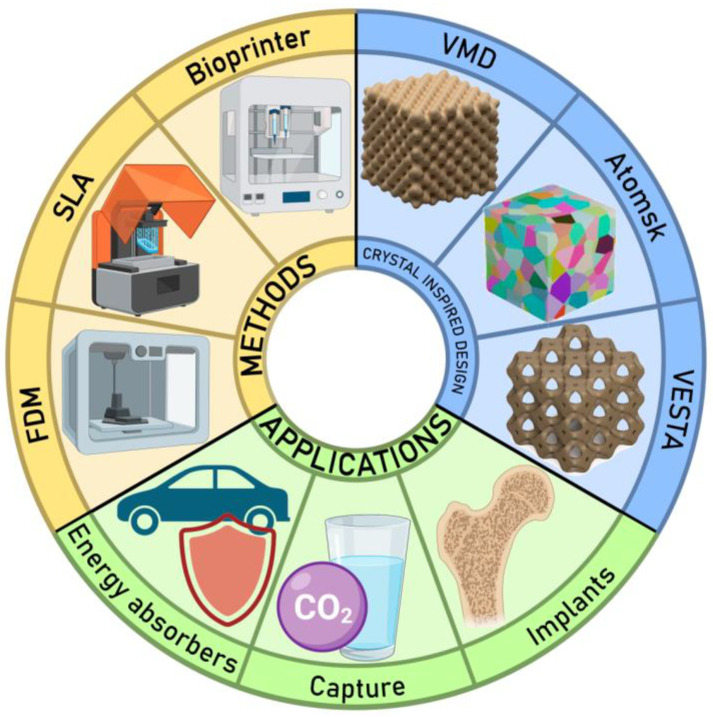
Schematic representation of cellular materials, their design methods, fabrication techniques, and applications. VMD is Visual Molecular Dynamics software 1.9.4 [32]; VESTA is Visualization for Electronic and Structural Analysis software3.5.5 [33], and Atomsk v0.13 is a tool to convert and manipulate atomic data files [34]. FDM and SLA are the AM and 3D printing techniques, fused deposition modeling (FDM) [35] and high-precision stereolithography (SLA) [36], respectively.

A schematic presenting general trends within the field of cellular materials is shown in Figure 2. Typical design methodologies for crystal-inspired materials involve such tools as Visual Molecular Dynamics (VMD) [32], which allows the design of crystal-inspired structures based on Gaussian density distributions to best replicate the crystal structure; Visualization for Electronic and Structural Analysis (VESTA) [33], which allows the construction of an isoelectronic surface based on electron density calculations to further design sheet-like crystal-inspired structures; or tools to mimic microstructure-like topology, such as Atomsk [34]. The most common synthesis approaches include AM and 3D printing techniques such as low-cost fused deposition modeling (FDM) [35], high-precision stereolithography (SLA) [36], and bioprinting [37]. Regarding applications, cellular structures with crystal-like geometries have been used as functional materials [38] for water and air purification [39,40], as implants [41,42], and as energy absorbers to protect objects from impact [43].

The current state of the research and key publications on crystal-inspired materials and TPMS-based materials are reviewed in this article. First, we review recent studies of crystal-inspired materials based on different classes of crystals. Then, we consider structural strut-based crystal-inspired materials.

## 2. Crystal-Inspired Lattices and TPMS

In the previous section, we discussed the potential advantages of TPMS-based materials, such as mass optimization (lightweight) and the avoidance of stress concentrations. Such properties are quantified mostly by mechanical properties (Young’s modulus, specific energy absorption, fracture strain, etc.). In this section, we review crystal-inspired metamaterials obtained from various classes of crystals (zeolites, schwarzites, and others) with an emphasis on their mechanical properties.

### 2.1. Zeolites

One type of crystalline material that is often studied is zeolites, a group of natural and synthetic aluminosilicate minerals that have a unique microporous structure. Zeolites are characterized by uniform pore size and large surface area, which makes them appropriate for such applications, including gas separation [44], catalyzing biomass conversion processes [45], and water treatment [46]. Besides that, zeolites have useful properties such as selective permeability and the ability to exchange ions [47,48,49,50]. However, the mechanical properties of zeolites are limited by their structural variety and high porosity.

**Figure 3:**
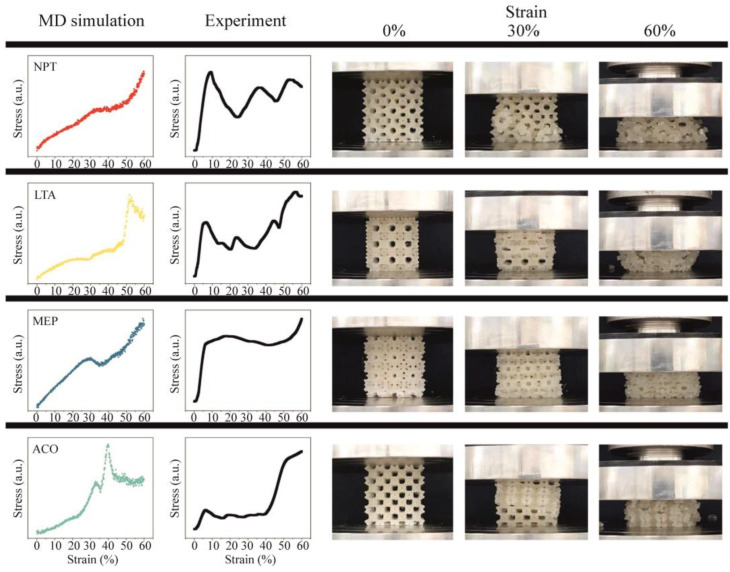
Results of compression test of 3D-printed (PLA) zeolite structure (red circles indicate initial fractures). To find out the abbreviation of zeolites, please refer to the IZA database [56]. The original work can be found at [51]. Reprinted with permission from Kim et al. [51], copyright © 2023 Copyright Clearance Center, Inc. (Danvers, MA, USA).

Kim et al. [51] investigated the relationship between the structural properties of zeolites at different length scales. In this study, 21 isotropic zeolite structures were chosen out of the 248 known structures. The zeolite samples were expanded to a 2 × 2 × 2 supercell and several of these structures were selected for compression testing. The similarity in the mechanical behavior of atomistic structures and of actual 3D-printed zeolite structures was observed. Besides that, the purpose of this study was to investigate the yield strength, elastic modulus, and energy absorption properties of the zeolite structures at micro- and macro-length scales. Molecular dynamics simulations were performed on 19 different cubic structures using the Large-scale Atomic/Molecular Massively Parallel Simulator (LAMMPS) [52]. The structural relaxation and deformation of silicon and oxygen zeolites were carried out using the reactive force field ReaxFF [53]. The behavior of the zeolite struts under investigation was isotropic, and therefore, compression was only applied in the Z-direction when testing 3D-printed structures. A compression deformation rate of 2 mm/minute was applied to 60% of the structure (Figure 3). Through a detailed study at the atomic scale, it has been confirmed that these structures can accurately replicate the results of experimental studies [23,54,55].

Kim et al. [51] found existing limitations in the approach used to compare MD modeling with 3D printing. Although the results obtained using TPU filaments showed a remarkable level of similarity, it is important to note that significant deviations under extensive deformation conditions occur between MD simulations and experimental results, primarily related to the occurrence of self-contact and densification. For some of the structures, the applied stress increased linearly as the deformation increased; for others, the response appeared to be non-linear. This suggests that the distinct structural features of zeolites, such as the distribution of pores and bond lengths, result in different mechanical properties. At strains above 40%, a rapid increase in the bond energy and a decrease in the angle energy lead to a significant increase in the stress. The 19 structures were divided into four classes based on their mechanical response (A, B, C, and D). Class A (CLO, NPT, JST, and JSR) exhibits a linear correlation between strain and stress, with a Pearson correlation index (R) greater than 0.95. To find out the abbreviation of zeolites, please refer to the IZA database [56]. In Class B (AST, ITV, LTA, RHO, RWN, and PWY), stress gradually increases up to approximately 40% of strain, after which there is a rapid increase in stress from 40% to 50%. For Class C (BV, FAU, MEP, MTN, SOD, and TSC), the inflection point occurs around 30% of the strain, where there is a decrease in stress followed by an increase. A strong correlation was observed between the density and the Si-O-Si bond angle of the structure, depending on compression stress, Young’s modulus, and energy absorption during reactive deformation. However, the Si-O bond length has less influence on mechanical characteristics compared to the Si-O-Si bond angle [57]. A correlation was also found between the characteristics of pores and the mechanical properties. The maximum value of stress that each structure can tolerate was closely related to the size of the pores. The results of this study could potentially provide valuable insights into the design and optimization of zeolite-based materials for diverse applications.

### 2.2. Schwarzites

Another type of frequently studied crystalline objects is 3D-printed schwarzite. Schwarzites consist of carbon nanostructures possessing the shape of TPMSs. Schwarzites are 3D crystalline allotropic carbon structures that were proposed in 1991 by McKay and Terrones [58,59,60,61]. Schwarzites have unique physical and chemical properties, such as high porosity and a large surface-to-volume ratio.

Herkal et al. [62] found that schwarzites have the property of energy dissipation/vibration isolation. The schwarzite geometry increases the damping coefficient. Also, biocarriers made on the basis of the schwarzite topology have optimal mechanical properties and increased efficiency in removing organics and nutrients from wastewater [39]. Multilayer 3D-printed structures on the basis of schwarzite with the formation of soft material in the areas of high-stress concentration significantly increase the specific yield strength and elasticity [63]. Another practical application of cellular materials can be as carriers for soft organic material, acting as a skeleton. For example, Daniel Saatchi et al. recently shared the sound and moisture absorption results of a multifunctional symbiotic lichen–Schwarz metamaterial, which uses Schwarz Primitive geometry as a framework, providing mechanical stability for organic lichen, which in turn has noise reduction and air humidification functions [64].

**Figure 4:**
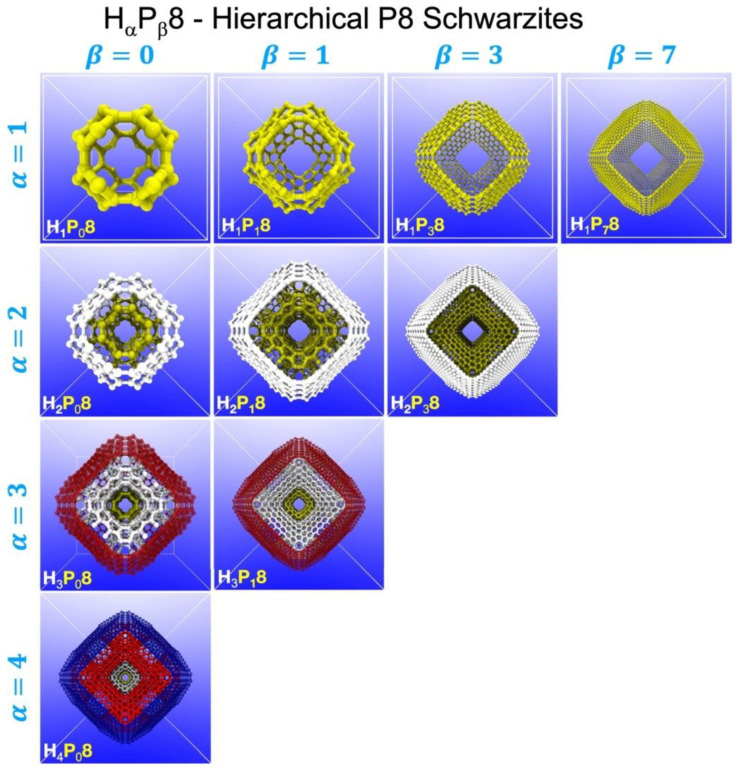
The petal-schwarzites structures investigated in the work of Bastos et al. [65], along with the system adopted to label them. The α value indicates the number of layers each structure possesses, while β indicates the smallest possible schwarzite present in its arrangements. The H4P08 structure, for example, has four layers, which means it contains all our four schwarzites, and the H1P08 one, in the form of the P80 unit cell, is the smallest structure present in it. Each layer was assigned a different color to better illustrate the concept: first layer (α = 1) is yellow, second layer (α = 2) is white, third layer (α = 3) is red, forth layer (α = 4) is blue. Reprinted with permission from [65], copyright © 2023 Copyright Clearance Center, Inc.

Bastos et al. [65] performed fully atomistic reactive molecular dynamics simulations to study the compressive properties of six newly proposed hybrid schwarzite-based structures (interlocked petal-schwarzites) (Figure 4). Experiments show a strong correlation between topology and mechanical properties, regardless of scale [55]. Due to their complex topology, unique distributions of stresses and strains occur. Six new petal schwarzite structures are created, with mechanical properties that exceed those of the original schwarzites. Stress–strain curves show the typical behavior of weak materials [66]. After an initial linear increase in compressive stress until the yield point, a plateau occurs, lasting until compaction, when there is a sudden increase in stress leading to the collapse of the structure. Schwarzites are highly resistant to compression and can be compressed up to 80% before breaking. In Table 1, the structural information of these schwarzites is presented. H1P78 has been identified as the most deformable. It can maintain its structural integrity up to 70% deformation, while accumulating very low levels of compressive stresses. Note that H1P08 and H1P18, which are denser than H1P38 and H1P78, have worse performance on the tests. The same trend is observed among the hybrid structures, as the denser ones were found to be less resistant.

The stress–strain curves obtained in that study can be divided into three regions. First (in the first region), petal-schwarzite structures behave in the same way. However, after a plateau for some structures, such as H2P08, H2P18, and H3P08, sharp changes in stress can be noticed. These fluctuations are caused by the early rupture of bonds, making these three structures less stable than the rest. The final stage of these petal-Schwartites occurs earlier. Compared to these three structures, the others can be compressed to higher deformation values without being destroyed. The petal schwarzite has the best properties of both the inner and outer layers, with higher yield and a densification region with high stress. It is important to note that, during the deformation of 60%, the curve for the inner layer shows destruction. At the same time, the curve of the petal schwarzite itself resists loading.

Based on the information provided, Bastos et al. [65] concluded that the strongest structure could withstand a deformation up to 90% of the initial sample height. The petal-schwarzite samples exhibit similar mechanical properties (maximum fracture strain/compressive strength) to the most durable variants of schwarzite. Petal-schwarzites also exhibit higher values of energy absorption per unit volume. All the structures studied present a positive coefficient of Poisson’s ratio. Furthermore, it can be deduced that as the number of interlocking layers increases, so does the mechanical resistance, and likewise, as the number of layers in the sample increases, so too does the mechanical performance. Experimental data have also shown that petal-schwarzite structures can absorb more energy before collapsing than pure schwarzites.

### 2.3. Pentadiamond

**Figure 5:**
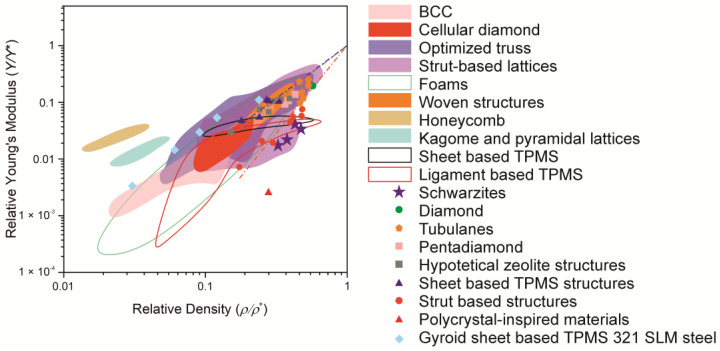
Ashby chart [69] for the relative Young’s modulus (Y/Y*) against the relative density (ρ/ρ*) on logarithmic scales for cellular structures with different topologies. Here, Y* and ρ* are, respectively, the Young’s modulus and density of the material investigated in the literature [9,67,70,71,72,73,74,75,76,77,78,79,80,81,82,83,84,85,86,87,88,89,90,91,92,93,94,95,96,97,98,99,100,101,102,103,104,105,106,107]. Type 321 steel is a standard austenitic 18/8 chromium nickel alloy steel. FDM is designated as a 3D printing method, fused deposition modeling [108].

Felix et al. [67] obtained excellent results investigating the mechanical behavior of atomic and 3D-printed pentadiamond models. Pentadiamond is a recently proposed carbon allotrope characterized by a covalent grid of pentagonal rings [68]. One of the key findings of this study was the observation that stress–strain behavior remains unchanged at different scales. The stress–strain curves of pentadiamond structures have four different areas: nonlinear behavior at low strain values, followed by linear behavior, quasi-plastic deformation, and, ultimately, compaction, leading to structural failure. Interestingly, it was found that Young’s modulus decreases with an increase in the number of pores in the material. The deformation mechanism mainly involved bending behavior, differing from the typical layered deformation mechanism seen in other 3D-printed structures. Moreover, these pentadiamond structures possessed exceptional energy absorption properties, and some even surpassed Kevlar. It is noteworthy that our analysis of the Ashby diagram (Figure 5) showed that the pentadiamond printed on a 3D printer exactly matches the ideal stretching and bending lines, which underlines their promise for applications requiring high energy absorption capacity. It follows from the figure that for TPMS structures, the sheet-based topology is more effective than the ligament-based one. Particularly notable are tubulanes, hypothetical zeolite structures, diamond, and pentadiamond, which have excellent mechanical properties. Schwarzites are somewhat inferior to this group of objects.

The research methodology included the manufacture of atomic and 3D-printed pentadiamond models followed by comprehensive tests to assess their mechanical characteristics. A comparative analysis of these models has shed light on their respective strength characteristics, providing valuable information about their performance characteristics. This study highlights the promising prospects for using 3D-printed pentadiamond structures for applications requiring high-energy absorption capacity combined with high strength and deformation resistance. These results pave the way for the development of advanced materials with exceptional mechanical properties and performance characteristics.

### 2.4. TPMS from Crystal Structure

**Figure 6:**
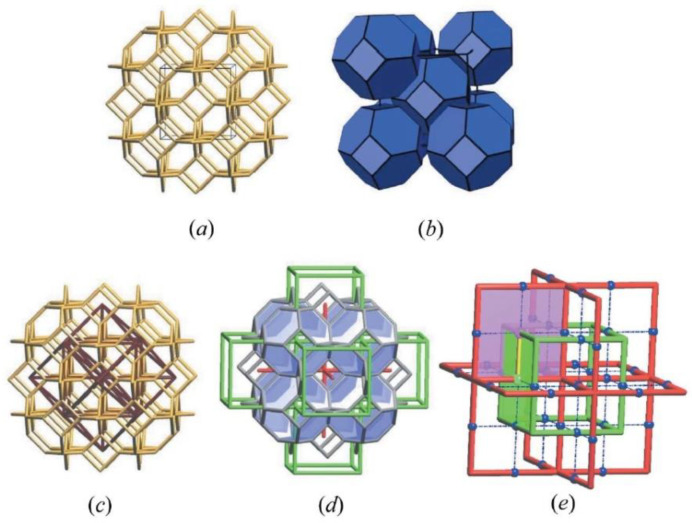
The framework of the zeolite sodalite (SOD): (**a**) the triply periodic net of the framework; (**b**) the natural tiling with tiles (truncated octahedra) separated for clarity; (**c**) the framework net (in yellow) together with the 14-coordinated extended body-centered cubic dual net bcu-x (in brown); (**d**) a triply periodic surface represented by blue facets (tile faces) constructed from tiling by removing the square faces of the natural tiles as well as two labyrinth nets (green and red) obtained by splitting the dual net and corresponding to two interpenetrating systems of channels separated by the surface; (**e**) Hopf ring net (blue balls and dotted lines), whose vertices coincide with the centers of square rings of the labyrinth nets and whose edges connect the centers of catenated rings. Two catenated rings are highlighted in red and blue, and the corresponding edge of the Hopf ring net is colored in yellow. Reprinted with permission from [109], Copyright © 2022 Peter Strikland, Executive Managing Editor, IUCr Journals.

Smolkov et al. [109] suggested a method of generating triply periodic surfaces that are isomorphic to a minimal surface from triply periodic crystal structures. They generated a triply periodic surface from the natural tilling of a crystal network by the removal of some tile faces and the smoothing of the resulting surface (Figure 6). After applying this method to all known zeolite structures, 98 triply periodic surfaces were obtained. Among these surfaces, there were 12 surfaces already known as TPMSs and four surfaces that could be characterized as isomorphic to new TPMSs. This showed that the method is simple and accurate for generating TPMSs from crystal structures.

### 2.5. Quartz

Markende et al. [110] described a new family of TPMSs: a QTZ-QZD family of surfaces, named for their parent networks, the quartz (qtz) network, and its double—the qzd network. The new QTZ-QZD family of surfaces is an irregular class of surfaces and has no in-surface symmetries; thus, it is difficult to generate by known methods [110]. The authors proposed an algorithm that can generate TPMSs with no in-surface symmetries based on their skeletal graphs. Their algorithm was a top-down approach coupled with a numerical construction in Surface Evolver and the complex analysis of minimal surfaces using Weierstrass–Enneper representation. The new QTZ-QZD family of surfaces obtained with a freely tunable pitch gives an opportunity to engineer on-chip chiral photonic devices such as beamsplitters or chemical sensors.

### 2.6. Liquid Crystals

Oka et al. [111] reported a method to solve the crystallographic phase problem of materials with a TPMS. Using the fact that the difference between the maximum and minimum electron densities tends to be smallest for the true phase combination among the possible combinations, a new algorithm for structure determination from diffraction data was developed. The method was tested on the bicontinuous cubic phase of lyotropic liquid crystals (LLCs) which has a TPMS topology. The authors proposed two indicators reflecting the plausibility of phase combinations of experimental data for the LLC bicontinuous cubic phase. The indicators were based on the structural features of materials. However, these indicators are only applicable to centrosymmetric space groups and become impractical with an increase in the number of independent reflections. This study emphasized that the new iterative algorithm opens the possibility of the application to structures without central symmetry, which tremendously widens the search space of phase combinations, thus overcoming previous difficulties. Using the described method, structure determination was achieved for all tested data sets. The obtained results were close to the true structures. The method appeared to be applicable independently of spatial resolution and the number of independent reflections and it is possible to determine a structure for which the space group is unknown. Also, this method could be applied to TPMS-like structures and such systems as three-dimensional periodic structures with bicontinuous or polycontinuous regions different from the TPMS-like structure.

### 2.7. Polymers and Elastomers

Sood et al. [112] studied the effect of material and lattice cells on static and dynamic mechanical properties of lattice cylinders from polylactic acid (PLA) and polyester elastomer (TPEE). They used the material extrusion (MEX) 3D-printing method. Due to possessing the same lattice structure, the cylinders of both polymers exhibited similar densification strains. PLA structural delamination caused the lattice holes to collapse, while TPEE failed due to the collapse of the connection holes.

### 2.8. High-Throughput Approach

**Figure 7:**
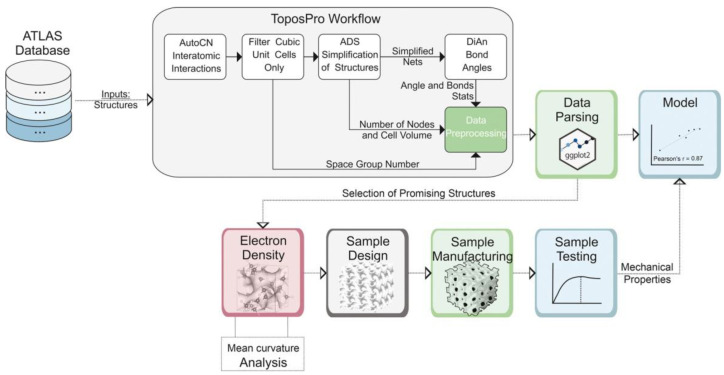
Algorithm of the crystallomorphic design of new cellular structures. Reproduced with permission from [99].

New high-throughput approaches are being developed to automatically find cells with the greatest strength, based on correlations of structural properties with the strength of a 3D-printed object, using databases of crystal compounds, as in our recently published work [99]. The Atlas of Prospective Zeolite Structures (ATLAS) database was used as an input [113]. The method is schematically presented in Figure 7. This approach allowed authors to find not only the most important correlations but also to discover completely new topologies, such as new cellular geometries with reinforced struts with increased strength and energy absorption. This property was discovered independently of other studies, in which cellular structures were developed using an explicit method [114]. This makes the suggested approach promising for creating new cellular structures with improved characteristics and predicting their properties.

## 3. Strut (Truss)-Based Crystal-Inspired Lattices

Nature-inspired periodic cubic lattices such as simple cubic (PC), volume-centered cubic (VCC), and face-centered cubic (FCC) lattices and others can act as structural materials. Libonati et al. [115] studied the mechanical behavior of cellular materials, additively fabricated using fused deposition modeling (FDM) [108], based on cubic Bravais crystals presented as beams with smoothed corners. They found that the printing direction effect, negligible in solid samples, becomes relevant in lattice structures, yielding different stiffnesses of struts and nodes. This phenomenon was accounted for in the proposed simulation framework. The numerical models of large arrays, used to define the scaling laws, suggest that the chosen topologies have a mainly stretching-dominated behavior—a hallmark of structurally efficient structures—where the modulus scales linearly with the relative density. By looking ahead, mimicking the characteristic microscale structure of crystalline materials will allow replication of the typical behavior of crystals at a larger scale, combining the hardening traits of metallurgy with the characteristic behavior of polymers and the advantage of lightweight architected structures, leading to novel materials with multiple functions.

Figure 4 shows the properties of strut-based lattices presented in the literature. It follows from the figure that strut-based topologies cover a wide range of Young’s modulus values, not inferior to other topologies. Recently, a new variety of such materials has appeared—polycrystal-inspired metamaterials [101,116,117,118,119,120,121,122]; however, from the figure, it follows that their properties are noticeably inferior to others. However, research in this area has only just begun, and the properties of grain boundaries are of fundamental interest. The use of metallurgical techniques allows flexible control of material properties.

## 4. Conclusions

**Figure 8:**
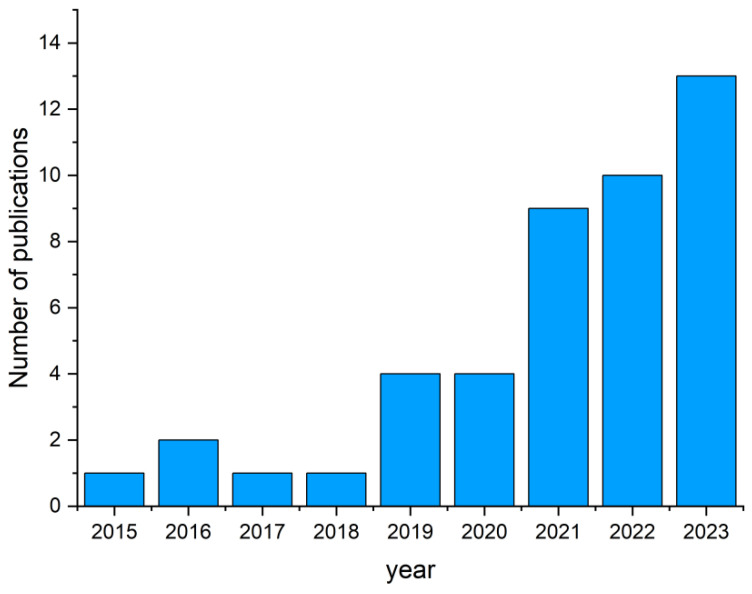
Research output in the realm of cellular metamaterials inspired by crystal structures.

TPMSs have significant potential for further research and development. An analysis of publication activity on this topic shows that active growth began five years ago. The search was carried out in the Google Scholar database using the “crystal-inspired” keyword (Figure 8). The topic remains rare, and articles are published in high-impact journals, including three articles already in *Nature* [101,117,123]. The most active areas are schwarzite, strut-based materials, and polycrystalline materials, which are currently receiving increased attention. In general, we can conclude that this is a highly promising, very young, and fast-growing field that requires special research skills at the intersection of sciences.

One can consider crystal-inspired materials as a new area of biomimetic materials. While inspiration is driven from non-living nature, there are many common features with traditional biomimetics with models being borrowed from nature and adjusted for engineering applications. A TPMS can also be used as a reinforcement of a composite material which may be filled with a liquid, forming a self-lubricating Slippery Liquid-Infused Porous Surface (SLIPS) [118].

The crystal-inspired approach is a powerful method for producing TPMSs and strut-based cellular materials for a wide range of functional purposes. Due to the variety of crystal structures, the variety of resulting topologies is unlimited. Methods for obtaining new topologies are constantly being improved. The properties of the resulting surfaces often exceed those of ideal TPMSs, and, for example, the mechanical behavior exhibits unusual behavior, both with and without correlation with the properties of crystals from the microscale. The presented review will help readers navigate the choice of methods for designing new surfaces for research on a new topic that is poorly studied and poorly represented in the literature. The performance of these metamaterials is characterized by their mechanical properties (Young’s modulus, the specific energy absorption, fracture strain, etc.). In the present work, we review crystal-inspired metamaterials obtained from various classes of crystals (zeolites, schwarzites, and others) with an emphasis on their mechanical properties.

## Figures and Tables

**Table 1:** Summary of the mechanical properties of schwarzites (*E* is Young’s modulus, *E/ρ* is the specific or gravimetric Young’s modulus, SEA is the specific energy absorption, *ε_F_* is the fracture strain, *S_E_* is the stroke efficiency, and *H* is the crush force efficiency). Based on [65].

Structure	E (GPa)	E/*ρ* (MJ/kg)	SEA (MJ/kg)	*ϵ_F_*	*S_E_* [%]	*H*
H1P08	116.0	57.54	21763	0.5	0.36	0.19
H1P18	71.86	62.22	44075	0.71	0.45	0.68
H1P38	30.17	48.19	14.68	0.86	0.49	0.76
H1P78	12.78	38.49	14.71	0.93	0.4	0.56
H2P08	86.21	45.95	34.18	0.84	0.39	0.29
H2P18	48.85	46.22	36.73	0.91	0.29	0.41
H2P38	19.14	31.12	44.22	0.95	0.44	0.6
H3P08	53.05	45.93	37.12	0.91	0.36	0.31
H3P18	24.52	36.49	45.87	0.96	0.38	0.42
H4P08	24.43	35.67	45.95	0.96	0.4	0.41
